# Co-Designing a Care Coordination Intervention for People With Motor Neuron Disease: Protocol for a Mixed Methods Study

**DOI:** 10.2196/96327

**Published:** 2026-08-04

**Authors:** Bryony Waters-Harvey, Kathleen Kane, Alys Wyn Griffiths, Grahame Smith, Clare M Bartlett, Lise Sproson, Sandra Smith, Jennie Starkey, Amy Clift, Theocharis Stavroulakis, Esther Hobson, Emily Mayberry, Alicia O'Cathain, Caroline Bidder, Katherine Kennedy, Jane Gibson, Christopher McDermott, Liam Knox

**Affiliations:** 1 Division of Neuroscience, School of Medicine and Population Health, Sheffield Institute for Translational Neuroscience (SITraN), University of Sheffield, 385a Glossop Road, Sheffield, England, S10 2HQ, United Kingdom, +44 1142222295; 2 School of Nursing and Advance Practice, Liverpool John Moores University, Liverpool, England, United Kingdom; 3 School of Clinical Dentistry, University of Sheffield, Sheffield, England, United Kingdom; 4 NIHR HealthTech Research Centre Long Term Conditions, Sheffield, England, United Kingdom; 5 Device for Dignity, NIHR HealthTech Research Centre in Long-Term Conditions, Sheffield Teaching Hospitals NHS Foundation Trust, Sheffield, United Kingdom; 6 Sheffield Teaching Hospitals NHS Foundation Trust, Sheffield, England, United Kingdom; 7 Sheffield Centre for Health and Related Research (SCHARR), University of Sheffield, Sheffield, England, United Kingdom; 8 Morriston Hospital, South Wales MND care and Research Network, Swansea Bay University Health Board, Swansea, Wales, United Kingdom; 9 Cornwall Partnership NHS Foundation Trust, Cornwall, England, United Kingdom; 10 Northern Lincolnshire and Goole Hospitals NHS Foundation Trust, Lincolnshire, England, United Kingdom

**Keywords:** motor neuron disease, MND, amyotrophic lateral sclerosis, care coordination, co-design, qualitative methods, health care, ALS

## Abstract

**Background:**

Motor neuron disease (MND), also known as amyotrophic lateral sclerosis (ALS), is a rapidly progressive neurological condition that requires complex multidisciplinary care. Within the United Kingdom, specialist centers provide expert interventions, while day-to-day support often relies on local nonspecialist community health and social care professionals. This is due to the distance between people’s homes and specialist centers, as well as the availability of specialist health and social care professionals. This can lead to fragmented communication and emotional, physical, and financial burdens, and it can be time-consuming for people living with MND, their carers, and the health care professionals involved in their care. Despite the recognized need for better care coordination, it remains inadequate in practice, with a current lack of specific evidence-based interventions for achieving this.

**Objective:**

The MND Together project aims to address these systemic gaps by (1) developing a national picture of care coordination in England and Wales, (2) identifying barriers and facilitators to coordination within specialist and nonspecialist settings, and (3) co-designing a practical care coordination tool with key stakeholders.

**Methods:**

This protocol outlines the co-design of an intervention underpinned by the Behavior Change Wheel and the Socioecological Model. First, a mixed methods, multicenter study will be conducted to develop a national picture, comprising focus groups with people living with MND, carers, and health and social care professionals. Second, focused ethnography will be conducted in 5 MND specialist centers and their catchment areas, involving 25 people living with MND, to explore the barriers and facilitators to coordination in practice. Finally, a series of co-design workshops will be conducted to identify key priorities for care coordination and to develop a new intervention, the MND Together tool.

**Results:**

The project started in September 2025 and will run until October 2027. Workstream 1 started in December 2025, with recruitment beginning at the first site in February 2026. To date, we have recruited 23 people living with MND and carers as well as 16 health and social care professionals across 4 of the 9 sites. Workstream 1 will end in August 2026, with results published at the end of 2026. Workstream 2 began in May 2026 and will run until February 2027, with results published in the summer of 2027. Workstream 3 will begin in March 2027 and conclude with the co-design intervention developed by late 2027. This will then be piloted in practice.

**Conclusions:**

By combining several methodologies with meaningful patient and public involvement and engagement, MND Together seeks to bridge the gap between specialist and community-based services. The MND Together tool aims to improve the quality of care and ensure that expert MND support is accessible as close as possible to every patient’s home.

## Introduction

### Background

Motor neuron disease (MND), also known as amyotrophic lateral sclerosis (ALS), is a progressive neurological condition characterized by the degeneration of motor neurons, leading to progressive muscle weakness in the limbs and affecting speech and swallowing, significantly impacting physical, emotional, and social functioning [1]. The prevalence of MND is approximately 8.58 per 100,000 [2]. It is a rapidly progressive condition, with a median life expectancy of 2 to 3 years from diagnosis, with death typically resulting from respiratory failure [3]. MND causes rapidly changing complexities; as the condition advances, the requirement for support grows exponentially [4]. Many individuals experience cognitive impairments—specifically executive dysfunction and language deficits—with a significant proportion developing frontotemporal dementia [5]. These combined physical and cognitive challenges heighten the need for specialist interventions. Consequently, the multifaceted nature of the disease necessitates a multidisciplinary approach to address its various clinical elements [4,6]. The team may include neurologists, physiotherapists, occupational therapists, specialist nurses, social workers, dietitians, speech and language therapists, respiratory nurses, and palliative care specialists [7].

While clinical practice guidelines advocate coordinated care delivered by specialist multidisciplinary teams to improve quality of life and longevity, the practical implementation of this model faces systemic challenges [6-9]. The relatively low prevalence of MND has necessitated a model in which patients travel to centralized specialist clinics staffed by a team of MND specialist health care professionals, but between appointments, they rely on nonspecialist community health and social care professionals for day-to-day support [10]. The geographic distance between specialist centers and community services, along with the involvement of multiple distinct organizations, often results in communication challenges and significant barriers to collaborative working. Uncoordinated care can impose emotional, physical, and financial burdens and can be time-consuming for people living with MND, their carers, and the health care professionals involved in their care [11,12]. Poor communication in MND care can result in organizations operating in isolation, unaware of who is responsible for specific patient needs [13]. Simultaneously, our patient and public involvement and engagement group, which we formed to support the development of this study, identified that the multitude of specialists involved can lead to role confusion, making it difficult for patients to navigate or remember their own care network. Furthermore, the relative rarity and challenges of MND often result in community-based health and social care professionals lacking the specialized knowledge required to manage complex interventions, such as gastrostomy, advanced care planning, and respiratory support [12,14]. Consequently, these practitioners rely on the expertise of specialist teams—support that is not always readily accessible. To ensure high-quality care, 2-way communication between MND specialist centers and nonspecialist community teams, patients, their families, and carers is required. Without seamless access to specialist expertise, there is a risk of poor care outcomes and increased emotional stressors for both providers and families [11,12]. These deficiencies are often most acute for underserved communities, including those in rural areas or those facing socioeconomic barriers to accessing centralized specialist services [15].

While previous research has established the urgent need for better care coordination, there remains a lack of specific, evidence-based interventions for achieving this within the current health and social care infrastructure. A coordinated care system would lead to better outcomes and care for people living with MND and their carers. The MND Together project aims to address this gap by co-designing a practical care coordination tool to improve the quality of care and ensure that expert MND support is accessible as close to every patient’s home as possible. This approach aligns with the strategic shifts outlined in the National Health Service (NHS) 10-year plan, specifically the transition from hospital to community [16]. The project will use a 4-workstream approach to co-design and evaluate this tool. This protocol will focus on the first 3 workstreams, which represent the development phase.

### Aims

This protocol outlines the following aims:

- Develop an understanding of current care coordination for people living with MND in England and Wales.
- Observe the barriers and facilitators of care coordination in MND specialist and nonspecialist community health settings across a range of services.
- Co-design the MND Together tool with stakeholders.

## Methods

### Overview

The development of the MND Together tool will consist of 3 workstreams, supported by the patient and public involvement and engagement group throughout. The first 2 workstreams will explore the current experience of care coordination for individuals living with MND. The findings from these workstreams will inform co-design workshops with stakeholders to develop a tool to address current challenges and barriers in care coordination. Reporting of the qualitative phases follows the SRQR (Standards for Reporting Qualitative Research) guidelines (Checklist 1) [17], while the patient and public involvement component is reported according to the GRIPP2 (Guidance for Reporting Involvement of Patients and the Public) checklist (Checklist 2) [18].

### Patient and Public Involvement and Engagement

A patient and public involvement and engagement group, comprising 12 people living with MND and carers, has been formed to collaborate with the researchers on all aspects of the study, including research design (recruitment strategies, topic guides, participant information sheets, and consent forms), research management, analysis and co-design, and dissemination. The group, co-chaired by the 2 lived-experience coapplicants, will shape and influence the study design throughout the course of the project. Working with people living with MND and carers, we will reflect on practical considerations and accessibility needs for conducting the interviews and focus groups to ensure these are as inclusive and impactful as possible. At the beginning of workstreams 1 and 3, equality, diversity, and inclusion workshops will be conducted with 4 underserved communities, including individuals from ethnic minority backgrounds, individuals who identify as LGBTQAI+ (lesbian, gay, bisexual, transgender, queer [or questioning], asexual [or allied], intersex), and individuals living in areas of recognized social deprivation, to gain insight into how the study can be adapted to reach individuals who may not participate in research due to barriers to access. The 2 lived-experience coapplicants of the research team will help co-develop the planning of the patient and public involvement and engagement group and the equality, diversity, and inclusion workshops.

### Ethical Considerations

Workstreams 1 and 2 received ethical approval from the NHS Cornwall and Plymouth (South West) Research Ethics Committee on December 12, 2025 (25/SW/0145). Ethics for workstream 3 will be submitted to the University of Sheffield Ethics Committee once workstreams 1 and 2 have been completed.

Informed consent will be obtained prior to participation. To accommodate varying accessibility requirements, informed consent will be obtained either in person or electronically. If a participant is not physically able to sign the consent form, they will be offered the option to verbally consent, with a witness present to verify their consent and sign on their behalf. There are information sheets and consent forms for each consent type. For recruitment completed at the study sites, consent will be conducted by the local NHS team or by the research and development delivery team. For recruitment through other methods, researchers will obtain consent. Consent is an ongoing process; the researchers will continue checking the participant’s consent throughout the study. Participants will be informed that they may withdraw at any time without providing a reason and that no further data will be collected from or about them; however, data already collected may be included in the final analysis. They will also be informed that withdrawal will not affect the care they receive. If participants take part in multiple workstreams, they will complete a consent form for each stream.

### Workstream 1

A multicenter, mixed methods study design will use focus groups to create a national picture of MND care in England and Wales and to understand how different services coordinate care to support people living with MND.

#### Recruitment

Participants will include (1) people living with MND and their carers, (2) former carers, and (3) health and social care professionals, including service managers and commissioners. We will collaborate with 9 NHS specialist MND centers across England and Wales to aid participant recruitment. The centers have been selected to provide a representative sample of different service setups and patient demographics, including rural versus urban locations, network services, and leadership styles.

People living with MND and their carers at the 9 MND centers will be invited to participate in the study by a member of their medical team, either in person or via phone. A total of 54 to 72 patients and carers (6‐8 participants from each site) will be recruited across the 9 centers. We will ensure that the sample comprises a range of disease severities, diagnostic dates, demographic characteristics, and treatments. Recruitment will also be conducted through Join MND Research (previously known as TiM-Research), an online research register for people living with MND across England and Wales [19]. Eligible registered users of the register will receive a digital invitation and the study information via their secure Join MND Research account. Interested individuals can then enroll directly by reviewing and signing an electronic consent form within the Join MND Research interface.

An additional 6 to 8 former carers of someone who had MND in the United Kingdom will be recruited through MND organizations and charities, such as the MND Association, the Darby-Rimmer Foundation, and the Ian Pratt Foundation.

At least 54 (6‐8 from each site) specialist and nonspecialist community health and social care professionals, NHS service managers (who oversee daily operations), and commissioners (who plan and fund local health care delivery) will also be recruited from the 9 centers. Information about the study will be sent to all specialist health care staff within the MND center via email, inviting them to participate. For nonspecialist community staff, contact details will be obtained from specialist centers if available. The research team will also search the internet for publicly available contact details for community staff at the NHS trusts where the center is located. Additionally, invitations will be sent to health and social care professionals with whom the research team has professional connections. The staff at each MND center will be asked to identify managers and commissioners. If the target is not met, further recruitment will be conducted through the MND Association and specialist mailing lists, such as the Neurology Academy.

All interested participants must be willing and able to participate in a focus group or interview. Individuals are eligible to participate if they are a person living with MND, a carer of someone living with MND, a former carer of someone who had MND, a health and social care professional, a service manager, or a commissioner. Health and social care professionals include people working in the health, social care, hospice, and charity sectors who have provided, or are currently providing, care to people living with MND. For managers and commissioners, eligibility applies to anyone involved in the organization of MND services. There are no exclusion criteria.

#### Procedures

Twenty focus groups will be conducted (2 per site and 2 additional online groups), each comprising 6 to 8 participants. Participants will be in a focus group with others similar to themselves, that is, people living with MND and carers together, or health and social care professionals together. To accommodate individuals’ accessibility needs, participants can share their experiences in alternative formats, such as individual or email interviews. Translators will be arranged as needed. To accommodate health and social care professionals, multiple shorter interviews and a range of group times will be offered.

Focus groups will discuss individuals’ experiences of care coordination between services. While the groups will focus on aspects of care that are important to participants, the questions will specifically address 4 areas of care in which coordinated delivery is crucial: gastrostomy, advance care planning, assistive living technologies, and respiratory support. People living with MND and their carers will be asked to discuss their preferences for care, self-help, and nonmedical interventions, as well as the barriers and facilitators to coordinating their care. Health and social care professionals will be asked to discuss their service organization, barriers and facilitators, the support provided to them, and their training needs. To protect participant privacy, all focus group data will be pseudonymized during transcription; however, while participants are requested to maintain the confidentiality of the session, absolute anonymity cannot be guaranteed within the group setting itself.

#### Analysis

Focus groups will be analyzed using a reflexive thematic analysis approach [20], facilitated by NVivo qualitative analysis software (Lumivero). An initial deductive approach will be adopted, using the Behavior Change Wheel’s Capacity, Opportunity, and Motivation (COM-B) categories [21] to group barriers and facilitators to care coordination within the Socioecological Model’s 5 stages [22]. An inductive analysis will then be conducted to develop the final themes, alongside feedback from our patient and public involvement and engagement group. The analysis will be conducted by 2 independent coders. To ensure conceptual alignment, the coders will initially analyze an identical 10% sample of the transcripts. They will then meet with the wider research team to discuss the codes, refine definitions, and agree on a collaborative coding framework. The primary coders will apply this framework to the remaining transcripts, and a second coder will independently audit an additional 20% of the transcripts. Disagreements or divergent interpretations will be resolved through reflective discussions and consensus meetings with the broader research team. The research team will also share the coding with the patient and public involvement and engagement group to ensure the interpretations align with their lived experience. The research team will actively engage in continuous reflexivity throughout the data collection and analysis phases. The coders, who have backgrounds in psychology and health and social care research, will maintain a reflexive journal to document their preconceptions, emotional responses, and how their backgrounds might influence data interpretation. Regular team meetings will explore assumptions to ensure the final themes genuinely reflect the participants’ experiences.

### Workstream 2

A focused ethnographic study will be conducted, comprising observations, interviews, reviews of medical records, and document reviews. Building directly on focus group findings, this stage will examine the interplay between barriers and facilitators that affect the coordination of care. By applying the Behavior Change Wheel [21], this study will conduct comprehensive behavioral system mapping and complete a behavioral diagnosis to identify systemic and individual factors influencing care coordination between specialist and nonspecialist teams.

#### Recruitment

Five of the 9 MND specialist centers from workstream 1 will be recruited. The challenges identified in the focus groups will inform the selection of centers, ensuring a range of services and catchment areas are represented. Twenty-five people living with MND and 25 carers (5 from each site) will be recruited. The recruitment process will follow the same procedure as workstream 1.

Participants with MND will be asked to identify any services they engage with that are separate from the specialist care centers (ie, community services). The research team will contact these additional services using publicly available details to obtain approval to conduct activities at the sites. All permanent and bank staff at the participating specialist and nonspecialist community services will be invited to participate. Health and social care professionals will be recruited through targeted emails to staff teams, research posters, and presentations at staff meetings. Sites will also be asked to identify managers and commissioners.

#### Procedures

Focused ethnography will be conducted to gain an in-depth understanding of practices, care coordination, and the complex associations between barriers and facilitators [23]. Adopting a nonparticipant observer stance, researchers will attend sites (clinical and domestic settings) to observe MND care coordination. Each person living with MND will be observed for a minimum of 6 hours as they engage with specialist and nonspecialist community services, including appointments, and community home visits. The research team will also observe interactions during multidisciplinary meetings where health and social care professionals discuss the care of participating patients. As with workstream 1, the ethnography will focus on the 4 aspects of MND care: gastrostomy, advanced care planning, assistive living technologies, and respiratory support. During the observation, information on the decisions made, the individuals involved, and the coordination and communication of those decisions will be collected. Throughout the observations, the researcher will engage in informal, in situ conversations before and after appointments and meetings to clarify the participant’s perspective on specific events or decisions.

Data will be collected through comprehensive written field notes recorded during the observations, and postobservation reflections from the researchers.

Patients’ medical records (where consent is obtained) and correspondence shared by the person with MND will be reviewed to gather information that cannot be obtained during observations and interviews, such as the number of appointments attended and the communication of decisions between teams.

All people living with MND and their carers, as well as 5 health care professionals per site (N=25) and 10 service managers, will be invited to participate in semistructured interviews to explore barriers and facilitators that are not observable. Interview techniques will be used to ensure that individuals with communication difficulties can participate, such as via email.

#### Data Analysis

Written ethnographic field notes and interview transcripts will be analyzed using ethnographically informed thematic analysis [24], managed within NVivo qualitative software (Lumivero). The researchers will use open coding to generate codes, which will be inductively developed into initial themes. These themes will then be refined and categorized through iterative discussions and constant comparative analysis both within and across the case study sites. The analysis will identify consistencies and divergences in patients’ journeys and the interplay between barriers and facilitators to care coordination, which will then be independently mapped onto the Behavior Change Wheel [21] and the Socioecological Model [22]. Each transcript or set of field notes will be analyzed by 2 coders. To ensure interpretative depth, both coders will independently code a subset of 20% of the combined field notes, interview transcripts, and medical notes. A shared coding framework will be developed with the wider team, and the coders will apply the framework to the remaining data. Disagreements will be discussed as a team and resolved through reflexive dialog and consensus meetings. Given the immersive nature of ethnography, the researchers will actively engage in continuous reflexivity to manage the dynamics by maintaining detailed reflexivity logs alongside their field notes, explicitly documenting their own emotional responses, their physical presence in the setting, and how their professional identity may influence participants’ behavior.

### Workstream 3: Coproduction Workshops

This workstream will comprise at least 3 rounds of co-design workshops with people living with MND, carers, health and social care professionals, and service managers to design a new tool or intervention, “MND Together,” to support care coordination between specialist and nonspecialist services for people living with MND [25]. The workshops will follow the Medical Research Council guidance [26] on developing complex interventions and use the Behavior Change Wheel [21] to select intervention functions to overcome barriers and support the facilitators highlighted in workstreams 1 and 2. The findings from the focus groups and focused ethnography will be brought together and shared with stakeholders to serve as the evidence-based foundation for the co-design workshops, where stakeholders will collaboratively translate identified barriers into the specific, practical features and user interface of the final MND Together tool.

#### Recruitment

Thirty participants will be invited to the workshops, comprising people living with MND, carers, health and social care professionals, and service managers. During workstreams 1 and 2, participants can express an interest in participating in workstream 3. Interested individuals will be emailed by the research team inviting them to participate in the workshops. If the target sample is not met among participants who have previously participated, the researchers will recruit participants through professional networks and MND organizations and charities, including the MND Association, Ian Pratt Foundation, and Darby-Rimmer Foundation.

#### Procedure

Participants will be invited to collaborate with other stakeholders in developing a tool or intervention in which all participants are equally involved and that addresses the group’s shared priorities and agreed-on goals. This approach uses integrated knowledge translation to ensure that those who will be using the tool are involved throughout the research process, increasing the likelihood of real-world impact and fostering a sense of shared ownership [25,27,28]. The rounds of workshops will be distributed evenly over 9 months. Each round may yield multiple workshops, depending on workload, participants’ needs, and emerging findings. Each workshop will last 2 to 4 hours (workshops may be split across multiple days) and will be conducted in person, online, or in a hybrid format, depending on participants’ requirements. Flexible methods will be offered, such as providing opportunities for individuals with communication difficulties to share written feedback using Post-it note activities. If written feedback is shared prior to workshops, it will be incorporated into the group discussions.

During the workshops, a range of participatory coproduction activities and techniques will be used, including vignettes to contextualize complex scenarios [29] and game-based elicitation tools, such as card-sorting exercises, to facilitate knowledge exchange and prioritize participant-driven outcomes. The activities and techniques will be selected based on participants’ needs, and alternative activities will be offered to accommodate accessibility needs. Participants may not attend all workshops. The 3 rounds of workshops are as follows:

- Round 1: ahead of the first round of workshops, participants will receive a booklet containing findings from workstreams 1 and 2. All participants will be invited to a workshop to discuss the findings from the previous workstreams and to identify the key challenges the MND Together tool should address. All participant groups will attend together, as advised by the patient and public involvement and engagement group and prior research, to jointly negotiate priorities between people with lived experience of MND and professionals [25,27,28]. The research team will present a recap of key findings, with a focus on barriers and facilitators, an overview of the mapped services from workstream 1, and intervention targets mapped to the Behavior Change Wheel and the Socioecological Model. Participants will be asked to review the data and discuss which intervention targets are most appropriate and will bring about change in care coordination. Intervention functions and targets will be guided by the results from the previous workstreams. Through small-group discussions, participants will select intervention functions and methods for their delivery, from the Behavior Change Technique Taxonomy, Behavior Change Wheel [30], and using the APEASE (Acceptability, Practicability, Effectiveness, Affordability, Side-effects, and Equity) criteria (Checklist 3) [30] as a guide. Participants will be asked to rate each intervention function and delivery method on affordability, practicality, effectiveness or cost-effectiveness, acceptability, safety, and equity in the context of NHS MND services. People living with MND and carers will be guided by the expertise of the researchers and health care professionals. The elements of the APEASE rating by participants will be determined by their prior experience.
- Round 2: depending on participants’ discussions in round 1, the second round of workshops may be conducted as a single session, or smaller workshops may be conducted based on participants’ agreed priorities. During the second round of workshops, participants will be asked to select behavior change techniques and delivery methods. When discussing behavior change techniques and delivery methods, participants will be asked to align the interventions with the APEASE criteria [30] to ensure that the final tool or intervention is as inclusive as possible. Disagreements among stakeholders will be resolved through group discussions. At the end of this round, the participants will reach a consensus on what the MND Together tool should be and include. Between rounds 2 and 3, the researchers will use the collected information and coordinate with the appropriate contacts to develop a prototype of MND Together. Such contacts will be determined by the specifics of the intervention, the finalization of which will follow the methods of co-design.
- Round 3: participants will be given the opportunity to engage with the MND Together prototype and provide feedback to further refine the tool. This testing and refinement are critical to ensure the tool is fit for purpose and addresses the needs identified by the stakeholders [31]. Participants will then be asked to co-design a logic model outlining how the tool’s components will produce short- to long-term outcomes. Finally, the group will identify the data to be collected in the fourth workstream of the project, a field-testing study. At the end of this round of workshops, the research team will use the feedback to finalize the MND Together tool in preparation for the field-testing study. Several workshops may be conducted to ensure sufficient time to test and refine the prototype.

#### Development of the MND Together Intervention

The MND Together intervention is being developed using an integrated coproduction framework. A core tenet of this methodology is that the specific typology of the intervention must emerge from the lived experiences of stakeholders (in this case, patients, carers, and multidisciplinary health care professionals) during the initial discovery phase. While the final form is not predefined, as the project aims to improve care coordination, including information sharing across multidisciplinary teams and across countries and trusts, it is likely that digital technology will play a role in the final MND Together intervention.

## Results

This project was funded by the NIHR Health and Social Care Delivery Research program in 2025. The project began in September 2025, with the 9 specialist MND centers being invited to participate in workstream 1 in December 2025. The first site started recruitment in February 2026. As of May 2026, four sites have been set up, with 23 people living with MND and carers, as well as 16 health and social care professionals, recruited across them. The study will continue until August 2026. A total of 66 to 88 people living with MND, carers, and former carers, along with 54 to 72 specialist and community health and social care professionals, NHS service managers, and commissioners, will be recruited across the 9 sites. The data will be analyzed and published in a peer-reviewed journal. The research team aims to publish the results from workstream 1 by the end of 2026.

Recruitment for workstream 2 opened in May 2026 and will run simultaneously with workstream 1. Currently, no participants have been recruited. Workstream 2 will end in February 2027. Overall, 25 people living with MND and 25 carers will be recruited. At this stage, we cannot determine how many health and social care professionals will be recruited, as this will depend on whom people living with MND interact with during observations. The researcher plans to obtain consent from all health and social care professionals who will be present for observations. Of the health and social care professionals recruited for the observations, 25 will be selected for interviews, along with 10 service managers. The data will be analyzed and published in several peer-reviewed journal papers. The research team aims to publish the results of workstream 2 in summer 2027.

Workstream 3 will begin recruitment in January 2027 and run until October 2027. Overall, 30 people living with MND, carers, health and social care professionals, NHS service managers, and commissioners will be recruited to participate in the workshops. The exact number of participants in each workshop will vary depending on participants’ requirements and the co-design process, but there will be a minimum of 15 participants per workshop. Workstream 3 will conclude with the development of the MND Together tool, which will be finalized in November 2027 in preparation for a field-testing study. The final architecture and functionality of the MND Together tool remain intentionally undefined at this stage. Its development is contingent on the synthesis of challenges and facilitators identified within the previous workstreams, ensuring the end product is empirically grounded in the findings of the co-design workshops. The co-design process and outputs will be reported in a peer-reviewed paper, which the researchers aim to publish in 2028.

Dissemination of all the workstreams will occur through conferences, the MND Together website, social media, and a summary of findings that can be disseminated through MND-associated organizations and charities.

## Discussion

### Anticipated Findings

This study will create a national picture of MND care services in England and Wales and develop a behavioral diagnosis of barriers and facilitators to care coordination between specialist and nonspecialist community services. The results from the workstreams will be mapped to the Behavior Change Wheel and Socioecological Model [21,22]. Workstreams 1 and 2 will identify individuals’ experiences of care coordination across NHS services in England and Wales and highlight barriers and facilitators to inform workstream 3 and the development of the MND Together tool. The results of this project aim to improve the quality of care delivered to people living with MND by enhancing coordination between MND specialists and community services through the use of the MND Together tool. The tool aims to provide expert MND care as close to each patient’s home as possible by improving communication and collaboration between MND experts and community teams.

### Strengths and Limitations

One of the strengths of this project is its multilens approach. By combining focus groups, focused ethnography, and the review of medical records and correspondence, the project ensures a comprehensive picture of care coordination in MND, ensuring that all elements can be considered, including policies and infrastructure. This is guided by the Medical Research Council guidance on developing complex interventions [26] and by well-grounded theories such as the Behavior Change Wheel and the Socioecological Model [21,22]. The research team prioritizes meaningful and inclusive stakeholder involvement throughout the project to ensure the research is accessible, and the MND Together tool is grounded in the needs of patients and carers and is implementable in real-world settings. The inclusion of equality, diversity, and inclusion workshops at 2 key stages of the project also aims to address challenges in reaching underserved groups, ensuring that the study design and recruitment account for the barriers to research access faced by these communities. Similarly, equality, diversity, and inclusion workshops during the co-design workstream will ensure that the final MND Together tool considers the inequalities faced by underserved communities in care coordination.

The complexity of coordination in MND services could make mapping all services engaging with people living with MND challenging, especially given that many people living with MND and their carers are unaware of which services the health and social care professionals are from. This indicates that the project may miss infrequent but critical care transitions. The design of the study requires the recruitment of NHS MND specialist centers which will act as sites and will complete recruitment and consent on behalf of the research team; therefore, the NHS R&D team has to agree on capacity and capability, which could lead to newer or smaller centers declining the invitation to participate. There is a potential for recruitment bias toward more well-established sites with strong teams that provide care coordination better than the UK norm. Similarly, the primary recruitment approach may miss individuals who have experienced the worst care coordination, as they may not engage with specialist centers. The research team has included recruitment via Join MND Research and MND-associated organizations and charities to reach these individuals. Additionally, recruiting former carers may capture experiences that might otherwise be missed through site recruitment.

### Conclusions

The MND Together project represents a significant step toward addressing the fragmented nature of care coordination for individuals living with MND in England and Wales. By exploring the current experiences of patients, carers, and health and social care professionals through focus groups and focused ethnography, this study aims to identify the barriers and facilitators affecting the delivery of care components, such as gastrostomy and respiratory support. The integration of the Behavior Change Wheel [21] and the Socioecological Model [22] will ensure the resulting intervention is empirically grounded and theoretically underpinned, targeting specific behavioral drivers within the NHS care system. Furthermore, the co-design approach in workstream 3 empowers stakeholders, including those from underserved communities, to act as coequal partners in designing a tool that is practical, equitable, and feasible for real-world clinical settings. Overall, this research seeks to bridge the gap between specialist centers and community-based services. By fostering improved communication and coordination, the MND Together tool will aim to ensure that expert, high-quality care is accessible as close to the patient’s home as possible, thereby enhancing the overall quality of life for those affected by MND.

## Supplementary material

10.2196/96327 Checklist 1 SRQR checklist.

10.2196/96327 Checklist 2 GRIPP2 checklist.

10.2196/96327 Checklist 3 APEASE criteria.

10.2196/96327 Peer Review Report 1 Peer review report by Health and Social Care Delivery Research program committee, National Institute for Health and Care Research (United Kingdom).

## Abbreviations

ALS: amyotrophic lateral sclerosis
APEASE: Acceptability, Practicability, Effectiveness, Affordability, Side-effects, and Equity
COM-B: Capacity, Opportunity, and Motivation
GRIPP2: Guidance for Reporting Involvement of Patients and the Public
LGBTQAI+: lesbian, gay, bisexual, transgender, queer (or questioning), asexual (or allied), intersex
MND: motor neuron disease
NHS: National Health Service
SRQR: Standards for Reporting Qualitative Research
