# Supplementary material for: Co-Designing a Care Coordination Intervention for People With Motor Neuron Disease: Protocol for a Mixed Methods Study
Source: JMIR Res Protoc. 2026 Aug 4;15:e96327. doi: 10.2196/96327 (PMC13436794; doi:10.2196/96327)
Supplement: Supplementary file 2 [file resprot-v15-e96327-s003.pdf]

Evaluation using the APEASE criteria: Acceptability, Practicability, Effectiveness, Affordability, Side-effects and Equity

Criteria	Description
**A**cceptability	Is the intervention acceptable to key stakeholders? If not, how can it be adapted to improve its acceptability?
**P**racticability	Can the proposed intervention be practically implemented, considering the available material and social resources?
**E**ffectiveness	Is it likely that the proposed intervention will be able to produce the desired effect or change within the target population? What is the time frame for this effect?
**A**ffordability	Can the proposed intervention be implemented in line with the available budget?
**S**ide-effects	Is it likely that the proposed intervention will lead to other outcomes, whether positive or negative, intended or unintended?
**E**quity	Is it likely that the proposed intervention will increase or decrease differences between advantaged and disadvantaged groups?

Michie S, Atkins L, West R. The Behaviour Change Wheel: A Guide to Designing Interventions. Silverback publishing; 2014:1003-1010. ISBN: 978-1-912141-04-3
