# Supplementary material for: Co-Designing a Care Coordination Intervention for People With Motor Neuron Disease: Protocol for a Mixed Methods Study
Source: JMIR Res Protoc. 2026 Aug 4;15:e96327. doi: 10.2196/96327 (PMC13436794; doi:10.2196/96327)
Supplement: Supplementary file 3 [file resprot-v15-e96327-s004.pdf]

NIHR | National Institute for Health and Care Research

NIHR Coordinating Centre
Alpha House
Enterprise Road
Southampton
SO16 7NS

Email: hsdrinfo@nihr.ac.uk

Dr Liam Knox
Research Fellow-Motor Neuron Disease
The University of Sheffield
Sheffield Institute For Translational Neuroscience
385a Glossop Road
England
S10 2HQ

28 January 2025

Dear Dr Knox

Reference: NIHR166956 MND Together: Improving communication and coordination of Motor Neuron Disease care

I am pleased to inform you that the Health and Social Care Delivery Research Funding Committee has recommended your application submitted for consideration in the call: **23/141 HSDR Researcher-led** for funding. The Department of Health and Social Care in its capacity as the National Institute for Health and Care Research (NIHR), has confirmed its intention to award funding upon acceptance of the terms and conditions set out in the Standard Research Contract and pending agreement to the suggested amendments recommended by the Funding Committee, as detailed in the enclosures.

The Standard Research Contract, between Contractors and the Secretary of State for Health and Social Care for all initiatives can be found on the NIHR website at:

https://www.nihr.ac.uk/researchers/i-need-help-designing-my-research/sign-a-contract.htm.

Next Steps

The NIHR is committed to the rapid initiation of research following the decision to fund to benefit members of the public and patients as soon as possible. Therefore, we expect funded researchers to be working towards gaining the necessary contractual agreements and governance approvals required to start the project within 8 months or by a date mutually agreed by both parties on acceptance of the award.

The NIHR acknowledges the risk to organisations around committing resource to research before a contract is in place; however, it is rare to not reach contractual terms unless the circumstance of the research team changes. The NIHR, therefore, encourages organisations to commit staff to setting up projects at as early an opportunity as possible in order to expedite the formal commencement of research.

It is acknowledged that there can be unforeseen delays in starting up a research project, but in order to help reduce these it is your responsibility to work closely with your organisation's R&D department or equivalent as well as other colleagues/departments involved in the administration and management of the research, and to start these discussions at the earliest opportunity.

To ensure that the project starts within the agreed timeframe with all the required agreements and approvals in place, appropriate staff (such as project and/or study managers) need to be in post as early as possible after receiving this letter of intent. These staff costs will ultimately be covered through the research funding award, but you are encouraged to meet them from Research Capability Funding (RCF) or equivalent prior to the research contract being agreed.

To support the often-iterative process towards agreement of the contract, we have set out the timeframes for the submission of responses or information for each step towards the agreement of the Standard Research Contract as well as the anticipated start date.

- Responses to Funding Committee feedback and queries **– 25 February 2025 (four weeks)**
- Responses to Finance and IP queries – **25 February 2025 (four weeks)**
- Contract signature **– no later than 1 month from the date the contract is issued for signature**
- Contracted commencement start **– no later than 8 months or a date mutually agreed on acceptance of award**

On receipt of information as set out above, the NIHR is committed to responding to your submission of information within two weeks or we will update you on progress.

The enclosures to this letter detail the feedback on your application, the processes to be undertaken during the next steps, and additional information relating to your award. Your contact person within NIHR is Hebe Stedman, who will be working with you on the contract. Please could you let Hebe know if you are unable to respond to the Funding Committee and Finance and IP queries by the deadlines noted above.

Yours sincerely,

Lynn Kerridge
Chief Executive Officer
NIHR Evaluation, Trials and Studies Coordinating Centre

Enclosures:
- Funding Committee, Finance and IP Feedback
- Research Management and Approvals Information

ENCLOSURE 1 – Comments and Feedback

Contact Details and Next Steps:

We understand that, in order for new studies to begin recruiting to their research team, details will need to be placed in the public domain (e.g. on websites) and discussions held with organisations supporting or involved in the study. However, aside from activities relating to recruitment of staff or project start-up we ask that you do not actively publicise or promote the award until the Research Contract has been fully signed.

Please complete your revised application in REALMS, including your responses to feedback (on the 'Response to Feedback' tab). Please also upload a revised detailed project description and submit the revised application back to us no later than **1pm on 25 February 2025.**

Going forward your contact person is Hebe Stedman, who can be contacted at postawardsetup@nihr.ac.uk.

Please log into your user account for the Research Awards Lifecycle Management System (REALMS) at https://realms.nihr.ac.uk/s_Login.jsp? to respond to the comments and requirements for changes. You will have access to the relevant parts of your application form to revise and resubmit with the required changes. Please note it is expected that this will be the final version of your application form, so please ensure that all justifiable relevant costs are included where appropriate.

As part of the submission, you should click on each feedback type on the 'Response to Feedback' tab of your revised application and respond to each comment in turn. If changes to your detailed research plan are required, you should also provide a revised copy of your plan which shows all the amendments as 'tracked changes' and which includes a version number and date. Please also upload a 'clean' copy of the plan with all tracked changes accepted.

These documents should be included as PDF uploads when submitting your revised application.

Now that your project has been recommended for funding (subject to a satisfactory response to the points raised in your intent to fund letter) we would like you to nominate signatories from your contracting institution who will approve the final revised application. Contact details need to be entered into the 'Other Supporting Roles - Signatories' section in the 'Project Contacts' tab of the revised application. All signatories need to be invited at the point of submission of your revised application.

Funding Committee Feedback

The following points of feedback should be responded to in the 'Response to Feedback' tab in your revised application in REALMS

1. The Committee welcomed this important research proposal focused on improving communication and coordination of Motor Neurone Disease care submitted by an experienced team. The Committee noted the team's robust, detailed and thoughtful response to feedback at Stage 1 and from external reviewers, with strengthened equity and diversity components and rebalancing of patient voice in the study design.
2. The Committee recommended this proposal for funding; however, the research team should consider the feedback below and make appropriate changes.

a. In recruiting people with lived experience, members asked the team to aim to capture range of severity of disease, time since diagnosis and other demographic variables within that.

b. In the case studies (WS2), the Committee members expressed concerns about the feasibility of carrying out 50 interviews and 150 hours of observation in twelve months by two researchers with only 5% FTE allocation of the WS lead, suggesting the team might need to review timing and resources in this component and potentially increasing the WS Lead's FTE to 10%.

c. In WS3, it would be challenging to ensure a diverse range of participants with a total sample of 30 to cover people with motor neuron disease, carers, healthcare staff, NHS managers and commissioners. The team may need to think about how this can be managed in order to have meaningful input, although noted that this co-design stage was well structured and specified.

d. Given the specialised nature of Motor Neurone Disease and the importance of including perspectives from patients, families, and carers, the Committee members suggested both increasing the number of patient and public involvement (PPI) members and the frequency of meetings to ensure comprehensive engagement.

e. The Committee members suggested expanding engagement with voluntary, charity and social care sectors.

f. PPI costings may be slightly under-estimated and should be reviewed.

g. The Committee members welcomed the inclusion of early evaluation in three real-world settings in WS4, suggesting that basic costings work could be added at this stage.

h. The Committee members would support a modest uplift in the research study budget to allow for the above activities, no greater than 5-10% of the total budget.

Finance Feedback

The following points of feedback should be responded to in the 'Response to Feedback' tab in your revised application in REALMS

1. 1.Can you confirm if all the research costs from SoECAT have been added to the application? If yes, could you please let me know under which section this has been added?

2. Please confirm the cost under other allowances mentioned for co-applicants from NHS Trust, does this include the Excellence Award? Please note that we pay for local awards, such as Local Clinical Excellence Awards. However, national awards are not allowed.

3. Please provide information/ details regarding the Access to 6 NHS-related databases through the Data Access Request Service (DARS).

4. Where costs are included for Researcher training, please advise the purpose of this training and confirm if this is specific to this research.

5. Please provide information/ details regarding the Neuroscience facility charge.

6. Can you confirm if the PPI costs under the PPI section are calculated in line with the NIHR INVOLVE guidance?

7. Please confirm the cost under other allowances mentioned for the staff from The University of Sheffield.

8. Please provide information/ details regarding the MND Together Development Costs, a breakdown of costs.

If you require any additional advice about the attribution of costs, please contact the finance team, email nets-finance@nihr.ac.uk. Please also refer to the AcoRD guidance available at: https://www.gov.uk/government/publications/guidance-on-attributing-the-costs-of-health-and-social-care-research.

Intellectual Property (IP) Feedback

In your revised application please use the 'Response to Feedback' tab to respond to standard IP questions relating to Background and Foreground IP.

If your application involves commercial partners, please ensure that they have had sight of the Research Contract template, specifically clause 16.5.

ENCLOSURE 2 – Research Management and Approvals Information

Research Governance

Guidance on the application process for ethical and other approvals can be found on the HRA website. Please note that if your study is led from England and involves the NHS in England you should apply for HRA approval.

If you are using patient information from an existing database, you should check whether the patients have given their consent for their data to be included in that database for research purposes, or if not whether the database is exempt under Section 251 of the NHS Act 2006. Where exemptions are not already in place, approval to use confidential patient information without consent must be requested from the HRA who make decisions with advice from the Confidentiality Advisory Group (CAG).

To take advantage of the growing utility of routine data (such as HES, GP records etc.), we would encourage investigators, where appropriate, to ask study participants to consent to long term follow up (e.g. beyond the outcomes to be collected in the funded trial) using routinely collected data, and appropriate linkage to allow this data to be best used.

Information on the responsibilities of key research stakeholders including Sponsors, Chief Investigators and Research Teams are summarised in the UK policy framework for health and social care research.

Research Delivery Network

The NIHR Coordinating Centre is a National Institute for Health Research (NIHR) non-commercial Partner. This means the studies that we fund may be eligible to access the NIHR Research Delivery Network (RDN) support, which is provided to health and social care research teams both in and outside the NHS in England. Where appropriate we expect you to apply for NIHR RDN support and subsequent inclusion in the NIHR RDN Portfolio of

studies to fully benefit from the support that the RDN offers through their Study Support Service. To find out more, please visit https://www.nihr.ac.uk/support-and-services/support-for-delivering-research/clinical-research-network/study-support-service and www.rdn.nihr.ac.uk/. If your study involves NHS sites in England you will need to apply for Health Research Authority Approval. For guidance on submitting an application please visit :www.hra.nhs.uk.

Clinical Trials Authorisation

You may require a Clinical Trial Authorisation from the Medicine and Healthcare products Regulatory Agency (MHRA). You should contact the MHRA and begin the application process. The MHRA website (www.mhra.gov.uk) has the latest up to date supporting information and guidance. The NIHR Clinical Trials Toolkit (www.ct-toolkit.ac.uk) also contains the latest information about Clinical Trials regulations and a helpful FAQ page.

Systematic Reviews Register - PROSPERO

Applicants undertaking systematic reviews should note the commitment of NIHR to publication in the PROSPERO database. PROSPERO was developed by the NIHR's Centre for Reviews and Dissemination (CRD), and is the first online facility to register systematic reviews for research about health and social care from all around the world. Access is completely free and open to the public. PROSPERO registration is a condition of NIHR funding for systematic reviews. Link to PROSPERO website: https://www.crd.york.ac.uk/prospero/.
